# Socializing practices of Irish children and adolescents with food allergy: A prospective study

**DOI:** 10.1016/j.jacig.2023.100164

**Published:** 2023-08-14

**Authors:** Miranda Crealey, Aideen Byrne

**Affiliations:** a Department of Paediatric Allergy, Children’s Health Ireland, Dublin, Ireland; b Department of Paediatrics and Child Health, Royal College of Surgeons in Ireland, Dublin, Ireland; c Department of Paediatrics, School of Medicine, Trinity College Dublin, Ireland

**Keywords:** Activities, adolescents, anaphylaxis, children, eating out, egg allergy, food allergy, restaurants, social

## Abstract

**Background:**

Food is an integral part of social activities; because of fear of accidental reaction, children with food allergy (FA) are at risk of exclusion or oversupervision at these events. The extent of adaptive exclusion behaviors is poorly defined. Families attending our service are encouraged to socialize and taught to minimize risk and avoid accidental reactions.

**Objective:**

The aim of this study was to establish the social practices and eating-out habits of children and adolescents with FA who are already attending an allergy clinic.

**Methods:**

Irish children, aged 2 to 16 years, with confirmed FA were recruited as part of a parallel prospective observational study titled Recording Accidental Allergic Reactions in Children and Teenagers (ReAACT). Information on social activities and eating out habits was collected prospectively.

**Results:**

A total of 531 children were enrolled. The majority attended age-appropriate social activities; 97% of the 5- to 12-year-olds went to birthday parties and 85% visited friends’ houses. More nonparticipators had previous anaphylaxis (relative risk [RR] = 1.44; 95% CI = 0.97-2.14; *P* = .06) and peanut allergy (RR = 1.19; 95% CI = 0.99-1.42; *P = .*06). Among adolescents, 94% visited friends’ homes, but only 12% had been away from home alone. Overall, 523 participants (98.5 %) visited food establishments, whereas 4.6% did not eat out in any food establishment; these participants were significantly more likely to be adolescents (RR = 3.27; 95% CI = 1.65-7.48; *P = .*0001).

**Conclusion:**

Overall, Irish children with FA are “living with allergy.” There was a trend toward decreased participation among adolescents. Future interventions should target this group specifically.

## Introduction

Many social events are marked by the preparation and sharing of food and customary treats. Food is an integral part of family celebrations (birthdays, christenings, and weddings) and cultural events (Christmas, Easter, Halloween, and Eid). Likewise, informal social gatherings commonly involve eating and often take place in food establishments (FEs) such as restaurants and cafes. Children with food allergy (FA) are potentially at risk each time they partake in food-related activities. Awareness of this risk creates anxiety for them and their parents or carers. Coping strategies can include avoidance, oversupervision, and restriction, even from events that are not particularly associated with food, such as sporting activities. Social engagement is integral to developing adaptive coping skills, social self-confidence, and healthy social relationships. Therefore, FA-related restrictions can have a negative impact on social functioning.^1^ A major goal of our allergy clinic is to empower the attending children and adolescents with FA to participate in all aspects of life (including social activities and eating out) just like their allergy-free peers.

The aim of this study is to report on the current social and "eating-out" practices of Irish children with FA who are attending our service. This study was supported by the National Children’s Hospital Fund (grant 15119).

## Results and discussion

This is the first large prospective study assessing the participation of children and adolescents with FA in social activities and their FE visitation habits over a 1-year period.

Most of the participants were male, and the median age was 7 years (interquartile range, 4-10 years); more than two-thirds of them had 2 or more FAs (Table I). Irish White race/ethnicity accounted for 85% of this population, which reflects the general Irish population (82.5% of which is of Irish White race/ethnicity).^2^

**Table I:** Baseline characteristics of participants

Characteristic	Participants (N = 531)
Sex, no. (%)	
Male	355 (67)
Female	176 (33)
Age at recruitment (y), median (IQR)	7 (4-10)
Group 1, no. (%)	141 (27)
Group 2, no. (%)	310 (58)
Group 3, no. (%)	80 (15)
Ethnicity and underrepresented populations, no. (%)	
White∗	485 (92)
(White Irish)	448 (85)
Underrepresented populations†	46 (8)
FAs, no. (%)	
1	174 (33)
≥2	357 (67)
Food allergen, no. (%)	
Cow’s milk	79 (15)
Hen’s egg	187 (35)
Peanut	339 (64)
Treenut	258 (48)
Fish	50 (10)
History of anaphylaxis, no. (%)	134 (25)
Allergic comorbidity, no. (%)	
Eczema	265 (49)
Asthma	221 (42)
Allergic rhinitis	330 (62)
No. of allergic comorbidities, no. (%)	
1	146 (27)
≥ 2	385 (73)
Environment, no. (%)	
Urban (city, town)	432 (82)
Rural (farm, isolated)	99 (18)
Family size (including child with allergy), no. (%)	
2	35 (6.6)
3-5	481 (91)
≥6	15 (3)
Single-parent household, no. (%)	35 (7)
If yes, single mother	31 (89)
Emergency accommodation at any time during the study, no. (%)‡	2 (0.3)
Diagnosis of autism spectrum disorder, no. (%)	9 (2)

*IQR*, Interquartile range.

Group 1 consists of children aged 2 to 4 years, group 2 consists of children aged 5 to 12 years, and group 3 consists of adolescents aged 13 to 16 years.

∗ White includes White Irish and White non-Irish individuals.

† Underrepresented populations refers to Black/Black Irish and Asian/Asian Irish individuals.

‡ Emergency accommodation refers to accommodation provided to homeless people.

Published standards for normal childhood participation in daily social activities are limited. The longitudinal Growing Up in Ireland cohort study found that three-fourths of 9-year-olds were involved in some form of organized sports club.^3^ According to non–peer-reviewed survey data, Irish primary school–age children (aged 5-12 years) receive an average of 8 to 12 birthday party invitations per year.^4^ In this study of children with FA, 302 (97%) of those aged 5 to 12 years, attend at least 1 birthday party annually, 272 (85%) visit friends’ homes, and 248 (80%) participate in extracurricular activities (Table II). Appropriately, the rate of parental supervision at birthday parties decreased from 100% in those younger than 5 years to 44% in those aged 5 to 12 years. The US National Confectioners Association reports that 93% of children go trick or treating at Halloween.^5^ Encouragingly, 91% of our 5- to 12-year-olds with FA also reported involvement in this door-to-door activity. Thus, for the majority of this age group, levels of social interaction reflect social norms. One area of low participation was overnight social activities, with only 10% of this age group sleeping at a friend’s house. A non–peer-reviewed survey of 1427 individuals reported that parents considered children aged 7 to 9 years mature enough for this activity^6^; therefore, the low level of involvement by the study cohort may represent use of an avoidance strategy by parents of children with FA; a comparison with the parents of their allergy-free peers would aid in interpretation of this number. Staying overnight involves more eating episodes than a party or playdate.

**Table II:** Participation of children with FA in social activities

Activity	Overall (aged 2-16 y) (N = 531)	Group 1 (aged 2-4 y) (n = 141 [27%])	Group 2 (aged 5-12 y) (n =310 [58%])	Group 3 (aged 13-16 y) (n = 80 [15%])
Parties, no. (%)				
Attends parties	439 (83)	89 (63)	302 (97)	48 (60)
Brings own food to party∗	183 (42)∗	44 (49)∗	123 (41)∗	16 (33)∗
Parents stay with child∗	225(51)∗	89 (100)∗	133 (44)∗	3 (6.5)∗
Friend’s house, no. (%)				
Visits friend’s house	387 (73)	46 (33)	272 (85)	69(94)
Eats food at friend’s house†	239 (62)†	30 (65)†	163 (60)†	46 (66)†
Parents stay with child†	88 (23)†	46 (100)†	39 (14)†	3 (4)†
Sleeps over at friend’s house	57 (15)	0	32 (10)	25 (36)
Disco, no. (%)				
Went at least once per year	34 (6)	0	20 (6.5)	14 (18)
Extracurricular activities, no. (%)				
Participated at least once per week‡	312 (80)‡	NA‡	249 (80)‡	63 (79)‡
Overnight trip/camp (without parents), no. (%)	10 (2)	0	0	10 (12)
Cultural celebrations				
Went “trick or treating” on Halloween	379 (71)	98 (70)	281 (91)	0

*NA*, not applicable as members of this group were not attending school.

∗ Denominator is children who attend parties.

† Denominator is children who visit friends' houses.

‡ Denominator is school-age participants.

A small number of children aged 2 to 4 years were attending friend’s houses, and 100% of them were supervised, as would be appropriate at this developmental stage.

In contrast to children aged 5 to 12 years, adolescents (aged 13-16 years) had lower levels of engagement in age-appropriate social activities. Although most of them (n = 69 [94%]) visit friends’ houses, only 25 (36%) had stayed overnight. Three families reported supervising their adolescents at friends’ homes or parties, which would not be conducive to normal social development. A minority of adolescents (n = 10 [12%]) had gone away from home alone and only 18% attended discos. Although this study did not examine the underlying causes of such choices, FA is likely to be a factor. A previous study found that adolescents with FA use avoidant coping strategies, leading to social impairment.^7^ Fewer adolescents reported attending parties (n = 48 [66%]), but this may be because parties are less commonly arranged in this age group. Reassuringly, 63 adolescents (79%) took part in extracurricular activities.

Among those children and adolescents who did not participate in any social activities (n = 47 [8.8%] [Table III]), there was a trend toward hen’s egg allergy (relative risk [RR] = 1.67; 95% CI = 0.95-2.9; *P = .*07), peanut allergy (RR = 1.19; 95% CI = 0.99-1.42; *P = .*06), and history of anaphylaxis (RR = 1.44; 95% CI = 0.97-2.14; *P = .*06). The association between hen’s egg allergy and no participation in social activities is a novel finding. Quality-of-life studies usually focus on peanut allergy and not on egg allergy because of its tendency to resolve. However, this study shows that in early childhood, when egg tolerance is low, egg allergy can be a barrier to normal social involvement, highlighting the importance of strategies to promote early resolution of egg allergy.^8^^,^^9^

**Table III:** Clinical characteristics of children with FA who are participating and not participating in social activities

Characteristic	Participates in ≥1 social activity (n = 484 [91%])∗	Do not participate in social activities (n = 47 [9%])	RR (95% CI)	*P* value
Age group, no. (%)				
Group 1 (aged 2-4 y)	131 (27)	10 (21)	0.74 (0.38-1.46)	.39
Group 2 (aged 5-12 y)	281 (58)	29 (62)	1.14 (0.65-2.01)	.62
Group 3 (aged 13-16 y)	72 (15)	8 (17)	1.15 (0.56-2.38	.39
Ethnicity and race, no. (%)				
White†	442 (91)	43 (91)	1.00 (0.91-1.09)	.97
Underrepresented populations‡	42 (9)	4 (9)	0.98 (0.36-2.6)	.98
No. of food allergies, no. (%)				
1	158 (33)	13 (28)	0.84 (0.52-1.36)	.49
≥ 2	326 (67)	34 (72)	1.07 (0.89-1.29)	.46
Type of allergy, no. (%)				
Cow’s milk	83 (17)	7 (15)	0.85 (0.38-1.8)	.69
Hen’s egg	226 (47)	29 (62)	1.67 (0.95-2.9)	.07
Peanut	304 (63)	35 (75)	1.19 (0.99-1.42)	.06
Treenut	230 (48)	28 (60)	1.25 (0.97-1.61)	.08
Fish	45 (9)	5 (11)	0.87 (0.36-2.0)	.87
Previous anaphylaxis, no. (%)	128 (26)	18 (38)	1.44 (0.97 to 2.14)	.06
Diagnosis of ASD, no. (%)	8 (2)	1(2)	1.28 (0.16-10.07)	.80

*ASD*, Autism spectrum disorder

∗ Social activities refer to going to parties, friend’s houses, or discos/socials.

† White refers to White Irish and White non-Irish individuals.

‡ Underrepresented populations refers to Black/Black Irish and Asian/Asian Irish individuals.

There is a paucity of studies reporting the eating patterns of children with allergy outside their home. This study provides novel insights into how families with FA balance risk and need for social engagement, with almost half of all participants (n = 183 [41.5%]) bringing food to parties and celebratory meals and slightly more than one-third (n = 148 [38%]) never eating food provided in a friend’s house (Table II). These results indicate awareness that the primary risk of severe adverse reaction is accidental ingestion rather than contact with environmental food particles and that providing food from home is preferable to preventing young people from socializing.

**Fig 1:**
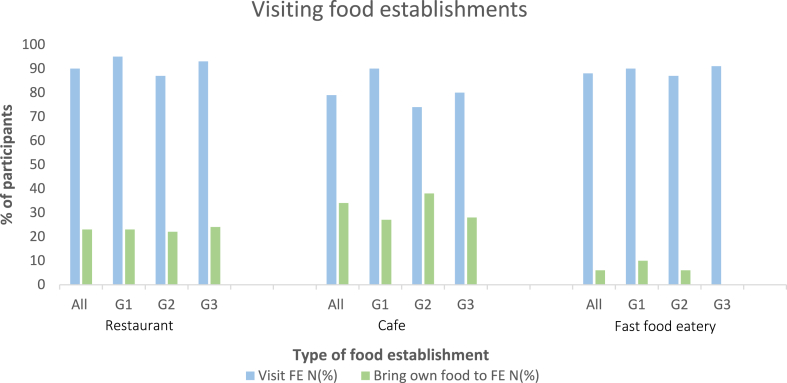
Children with FA visiting FEs. All refers to all participants, G1 refers to children aged 2 to 4 years, G2 refers to children aged 5 to 12 years, and G3 refers to children aged 13 to 16 years.

Minimal data exist on the eating behaviors and FE choices of children and adolescents with FA. Almost all of this study’s participants (n = 523 [98.5%]) visited at least 1 type of FE accompanied by parent or caregiver annually; restaurants were visited by 90% and fast food eateries were visited by 88%. Significantly fewer participants visited cafes (79% [95% CI = 73.3-88.9; *P* < .005]) (Fig 1). Half of the participants (n = 262 [50%]) did not restrict the type of FE visited and ate in all 3 types of FEs. The median frequency of visiting an FE was once per month (interquartile range, once every 3 months to once per week). Only half of the adolescents (n = 40 [50%]) visited FEs without a parent or caregiver. There are no comparable data in the literature; however, it is reported that families with FA seek familiarity in choosing FEs.^10^, ^11^, ^12^ In this study, more than three-fourths eat in only FEs that they had previously frequented. This strategy may bring a false sense of safety, with families less on their guard if they have had a previous positive experience, although it should be acknowledged that some FEs are better at managing FA.

Overall, a quarter of participants (n = 108 [23%]) who visited restaurants brought their own food (Fig 1). There was a significant difference between the number of children bringing their own food to cafes and the number of those bringing their own food to restaurants (an 11% difference [95% CI = 5.08-16.86; *P = .*003]), a significant difference between the number bringing their own food to restaurants and the number bringing their own food to fast food eateries (a 15% difference [95% CI = 11.9-20.6; *P < .*05]), and a significant difference between the number bringing their own food to cafes and the number bringing their own food to fast-food eateries (a 17% difference [95% CI = 12.65- 21.36; *P* < .005]). These data provide further insights into families' coping mechanisms. The data show that these families of children with FA are risk-stratifying, perceiving that fast food restaurants are safer. Data from Recording Accidental Allergic Reactions in Children and Teenagers (ReAACT) indicate that FEs are the second most common location (n = 36 [16%]) of accidental reactions after the home.^13^ Eight reactions occurred in fast food eateries despite parents considering them a “safer” option. Oriel et al also found fast food eateries to be a frequent site of reactions at FEs.^14^ Differential risk perception is further displayed by the evidence that more participants (as many as two-thirds) bring their own food to friends’ houses and parties than to restaurants. This has not been reported previously.

Furthermore, by bringing their own food to FEs, one-fourth of those studied do not get the experience of eating out or learn to read ingredient lists and make ordering decisions. Indeed, communication within an FE was concerning in this group; the rate of checking allergen information was less than 100% (n = 405 [87%]), and only 65% of participants in this group (n = 338) informed FE staff of their child’s FA.

A small number of participants (from the group aged 5 to 12 years and that aged 13-16 years) (n = 26 [4.9%]) did not visit any FE. These children were significantly more likely to be adolescents (RR = 3.27; 95% CI = 1.65-7.48; *P = .*0001) and have a hen’s egg allergy (RR = 1.9; 95% CI = 1.31-3.00; *P = .*001). There was also a trend toward having a history of anaphylaxis (RR = 1.76; 95% CI = 0.79-3.94; *P = .*163) and 2 or more FAs (RR = 1.21; 95% CI = 0.99-1.48; *P = .*06) in this group (Table IV). It is concerning that adolescents were 3 times less likely to eat in FEs than younger participants. These figures likely reflect avoidant behavior rather than cultural norms. The Irish National Food Consumption Survey 2020 revealed that for adolescents without an allergy (aged 13-18 years), 20% of their calories come from food prepared outside the home.^15^ This avoidant behavior contrasts with the risk-taking behaviors in adolescents reported in other studies.^16^^,^^17^

**Table IV:** Clinical characteristics of children eating in FEs and children not eating in FEs

Characteristic	Eats food at ≥FE (n = 505 [95%])	Does not visit or eat in any FE (n = 26 [5%])	RR	95% CI	*P* value
Age group, no. (%)					
1 (aged 2-4 y)	141 (28)	0	0.07	0.004-1.03	.05
2 (aged 5-12 y)	294 (58)	16 (62)	1.05	0.77-1.44	.73
3 (aged 13-16 y)	70 (14)	10 (38)	3.27	1.65-7.48	**.001**
Ethnicity and race no. (%)					
White∗	462 (95)	23 (5)	0.97	0.83-1.11	.64
Underrepresented population†	43 (93)	3 (7)	1.36	0.42-4.40	.536
No. of food allergies, no. (%)					
1	169 (33)	5 (19)	0.58	0.26-1.28	.17
≥ 2	336 (67)	21 (81)	1.21	0.99-1.48	.06
Type of allergy, no. (%)					
Cow’s milk	32 (12)	1 (6)	0.6	0.08-4.27	.61
Hen’s egg	127 (25)	13 (50)	1.9	1.31-3.00	**.001**
Peanut	323 (64)	16 (62)	0.96	0.70-1.31	.80
Treenut	248 (49)	10 (38)	0.78	0.48-128	.78
Fish	49 (10)	1 (4)	2.5	0.36-17.56	.35
Diagnosis of ASD, no. (%)	9 (2)	0	1.1	0.07-17.13	.93
Previous anaphylaxis, no. (%)	125 (25)	9 (35)	1.39	0.79-2.42	.23

*ASD*, Autism spectrum disorder.

*P* value less than .05 was deemed significant and is highlighted in bold.

∗ White refers to White Irish and White non-Irish individuals.

† Underrepresented population refers to Black/Black Irish and Asian/Asian Irish individuals.

The strength of this large study is its provision of unique data on the day-to-day social and food consumption habits of a well-defined cohort of young people with FA.

The study's limitations include lack of measurement of the quality of the social experience. In addition, this study provides data on adolescents only up to the age of 16 years. The study findings may be unique to the Irish population, and therefore, similar studies should be replicated in other jurisdictions. The findings cannot be applied to populations that have not had the opportunity to attend an allergy clinic in which they have had education on managing FA. We acknowledge that there are more complex issues to consider other than just whether children engaged socially. We did not explore any adaptions that might be necessary to allow the child with FA to socialize or whether there were specific restrictions to socializing (eg, attending only a particular friend's house and eating 1 particular food). This warrants further research. In addition, there was a paucity of normative data with which to compare our cohort.

It is imperative that we use the positive findings in this study to benefit the wider paediatric population with FA. The results of this study prompt a number of recommendations, including the following: (1) encourage identification of children with FA and restrictive patterns of socialisation as part of allergy clinic assessments, and (2) target adolescents (eg, group educational sessions could be an initial step in supporting this group to both visit FEs and communicate with staff while there). In addition, future research should investigate how children with FA and their families navigate social situations.

In conclusion, here we have reported that children with FA who are attending an allergy clinic are “living with allergy”; they are participating in social activities and eating out in FEs. There was a trend toward decreased participation in adolescents.

For detailed methods, please see the Methods section in this article's Online Repository at www.jacionline.org.

## Disclosure statement

Supported by the National Children’s Hospital Fund (grant 15119).

Disclosure of potential conflict of interest: The authors declare that they have no relevant conflicts of interest.

**Key messages**

• Children with food allergy do partake in age-appropriate social activities with their allergy-free peers.
• Lack of social participation is associated with hen's egg allergy and previous anaphylaxis.
• There is a risk of decreased social participation among adolescents. Clinicians should address this risk in the clinic setting to support normal social development.
