# Supplementary material for: Socializing practices of Irish children and adolescents with food allergy: A prospective study
Source: J Allergy Clin Immunol Glob. 2023 Aug 14;2(4):100164. doi: 10.1016/j.jacig.2023.100164 (PMC10509833; doi:10.1016/j.jacig.2023.100164)
Supplement: Supplementary data [file mmc1.docx]

Data for online repository

**Methods**

All participants in this study were recruited as part of a parallel prospective observational study, “Recording Accidental Allergic reactions in Children and Teenagers” (ReAACT). Enrolment was from the cohort of food allergic children attending the 2 allergy services at Children’s Health Ireland (CHI). The inclusion criteria were: (i) Age >2 yrs <17yrs of age (ii) a diagnosis of IgE mediated food allergy (FA).

Participants were defined as having a diagnosis of IgE mediated FA if either of the following 4 criteria were met at study entry: (i) a clear history of a recent reaction (previous 6 months) clinically consistent with immediate IgE mediated allergy and a positive skin test >3mm or (ii) a history of a reaction in the past (not necessarily the recent past) and a skin test in the past 6 months to that allergen, of >7mm or (iii) a positive OFC performed at CHI Tallaght or CHI Crumlin in the past 6 months or (iv) a history of a reaction in the recent past (previous 6 months) and evidence of sensitisation to a non- specific lipid storage protein (LPS). This strict study inclusion criteria ensured that participants were still food allergic at study entry.

The exclusion criteria were: (i) < 2years or >16 years (ii) an isolated diagnosis of non-IgE mediated FA (e.g. Eosinophilic oesophagitis or food protein induced enterocolitis) or pollen food syndrome, (ii) Cow’s milk (CM) and/or hen’s egg allergy on the IFAN cow’s milk or hen’s egg ladder without evidence of a recent reaction, (iv) participation in another research study, (v) a diagnosis of a condition that can be difficult to differentiate from acute allergic reactions such as Physical or Idiopathic Urticaria, Cutaneous or Systemic Mastocytosis, very severe or difficult to control atopic dermatitis, (vi) children with a significant or complex medical history such as cystic fibrosis or developmental delay. Children with complex medical history were excluded as their social activities may not have been representative of the general allergy population. Children with chronic conditions have been shown to have decreased social functioning^1^. In addition, they may have excessive levels of supervision not in keeping with other children of their age.

Participants were recruited over 7 months (Nov 2018-May 2019) into 3 age subgroups: group 1 (G1), 2-4 years old, group 2 (G2), 5-12 years old, group 3 (G3), 13-16years old; both new and return patients were recruited. Participant’s involvement in social activities and eating out habits were assessed by interview with parent +/- patient, using a 42-item baseline questionnaire at recruitment. As part of ReAACT, contact was made with participants every 3 months by phone for a year to assess for accidental allergic reactions. They were also asked every 3 months what their current activities and eating out patterns were, to allow for the prospective gathering of this data.

Extracurricular activities were defined as any activities taking place outside of school and included team sports, tennis, swimming, gymnastics, athletics, horse riding, dancing, art, music, scouts.

The term FE was used to describe all places where food was bought and consumed: restaurants (included standalone restaurants and restaurants within hotels, pubs) cafes (including coffee shops), fast-food establishments (including takeaways).

Participants defined for themselves the type of FE with the above guidance from the researcher.

Visit to a friend’s house was defined as visiting the house of a peer. Attendance at a party was defined as visiting a house/venue for another child’s birthday party.

“Trick or Treating” is a Halloween ritual custom where children in costumes travel from house to house, asking for treats with the phrase "Trick or treat". Typically, it is younger children that partake. The "treat" is traditionally peanut in the form of a “monkey nut” or confectionary. It is very popular in Ireland. Easter is another cultural holiday in Ireland. At Easter time, Irish children traditionally receive a chocolate egg from a folklore figure “the Easter Bunny”.

Ethnicity, race, and gender were self-reported.

Participants with a formal diagnosis of autism spectrum disorder were identified; ASD is associated with socialisation and feeding difficulties. Ethics approval was received from the Research Ethics Committees (REC) in CHI (REC Reference: GEN/672/18).

SPSS (version 27: 2020; SPSS Inc., Chicago, IL, USA) was used for analysis. Demographic and clinical characteristics were compared using two-sample t-tests or Wilcoxon rank-sum tests for continuous variables and Fisher’s exact or chi-squared tests for categorical variables. For normally distributed data, mean and standard deviation was reported. For data with a skewed distribution, median, lower and upper quartiles were reported. To ensure a high level of accuracy, confidence intervals were set at 95% and the study was powered at 80%. A P < 0.05 was considered statistically significant. Proportional differences between categorical variables were calculated by using relative risks (RR).

**References**

**E1.** Adams CD, Streisand RM, Zawacki T, Joseph KE. Living With a Chronic Illness: A Measure of Social Functioning for Children and Adolescents. *Journal of Pediatric Psychology.* 2002;27(7):593-605.
